# Determinants of the level of circulating-tumor HPV16 DNA in patients with HPV-associated oropharyngeal cancer at the time of diagnosis

**DOI:** 10.1038/s41598-023-48506-6

**Published:** 2023-12-01

**Authors:** Marek Kentnowski, Alexander J. Cortez, Agnieszka M. Mazurek, Jolanta Mrochem-Kwarciak, Anna Hebda, Urszula Kacorzyk, Katarzyna Drosik-Rutowicz, Ewa Chmielik, Piotr Paul, Karolina Gajda, Izabela Łasińska, Barbara Bobek‑Billewicz, Andrea d’Amico, Krzysztof Składowski, Mirosław Śnietura, Daniel L. Faden, Tomasz W. Rutkowski

**Affiliations:** 1 Maria Sklodowska-Curie National Research Institute of Oncology Gliwice Branch, Wybrzeże Armii Krajowej 15, 44-101 Gliwice, Poland; 2 Department of Medical and Experimental Oncology, Cancer Institute, Poznań University of Medical Sciences, 16/18 Grunwaldzka Street, 60-786 Poznan, Poland; 3 Department of Nursing, Institute of Health Sciences, University of Zielona Góra, 2 Energetyków Street, 65-417 Zielona Góra, Poland; 4 Department of Pathomorphology and Molecular Diagnostics, Faculty of Medical Sciences in Katowice, Medical University of Silesia, Katowice, Poland; 5 Department of Otolaryngology-Head and Neck Surgery Harvard Medical School, Mass Eye and Ear, Mass General Hospital, Broad Institute of MIT and Harvard, Cambridge, USA

**Keywords:** Cancer, Molecular biology, Molecular medicine, Oncology

## Abstract

Circulating tumor HPV DNA (ctHPV16) assessed in liquid biopsy may be used as a marker of cancer in patients with HPV-associated oropharyngeal cancer (HPV + OPC). Factors influencing the initial ctHPV16 quantity are not well recognized. In this study we aimed to establish what factors are related to the level of ctHPV16 at the time of diagnosis. 51 patients (37 men and 14 women, median age of 57 years old) with HPV + OPC prior to definitive treatment were included. ctHPV16 was measured by qPCR. Tumor and nodal staging were assessed according to AJCC8. Blood derived factors included squamous cell carcinoma antigen (SCC-Ag), serum soluble fragment of cytokeratin 19 (CYFRA 21-1), C-reactive protein (CRP), albumin level (Alb), neutrophils (Neut), thrombocytes (Plt) and lymphocyte (Lym) count, Neut/Lym ratio were assessed. The volumes of the primary tumor (TV) and involved lymph nodes (NV) were calculated using MRI, CT or PET-CT scans. Data were analysed using parametric and nonparametric methods. Variables for multivariable linear regression analysis were chosen based on the results from univariable analysis (correlation, univariable regression and difference). There were 9 (18%), 10 (19%) and 32 (63%) patients who had TV and NV assessed in MRI, CT or PET respectively. Primary tumor neither as T-stage nor TV was related to ctHPV16 level. Significant differences in the ctHPV16 between patients with high vs low pain (P = 0.038), NV (P = 0.023), TV + NV (P = 0.018), CYFRA 21-1 (P = 0.002), CRP (P = 0.019), and N1 vs N3 (P = 0.044) were observed. ctHPV16 was significantly associated with CYFRA 21-1 (P = 0.017), N stage (P = 0.005), NV (P = 0.009), TV + NV (P = 0.002), CRP (P = 0.019), and pain (P = 0.038). In univariable linear regression analysis the same variables predicted ctHPV16 level. In multivariable analyses, CYFRA 21-1 and CRP (both as categorical variables) were predictors of ctHPV16 level even above NV. ctHPV16 at presentation is driven by tumor volume measured mostly by N. CYFRA 21-1 and CRP are additional factors related to ctHPV16 prior to the treatment.

## Introduction

The number of patients suffering from HPV-associated oropharyngeal cancer (HPV + OPC) is increasing ^1–3^. Despite a better treatment response for HPV + OPC patients compared to non-HPV-associated head and neck cancers, no treatment de-escalation has been approved ^4^ and nearly 25% of patients experience treatment failure ^5,6^. Circulating tumor HPV DNA (ctHPV16) in plasma has been shown to be a highly accurate real-time biomarker of HPV + OPC, reflecting the presence, absence and changes in cancer burden across time ^7–11^ not only in the relation to the volume of disease but probably also to dynamics of tumor cell proliferation and mechanisms of cell death due ^12^. ctHPV16 may be used as a promising biomarker for early detection of HPV + OPC ^9,13,14^, to support monitoring of treatment response during therapy ^11,13,15,16^ and shortly after its completion ^17^ but also during surveillance to indicate disease recurrence ^18,19^. In such cases, the initial amount of ctHPV16 as a reference value may be of clinical significance ^11,20^. Some datasets suggests that higher amount of initial ctHPV may be a positive prognostic factor, while others have found no association, or even the opposite relationship^21^. Little is known about factors which are linked to initial ctHPV16 level although recent work suggest nodal volume may be a driving force ^22^. In this study we examined selected tumor and patient related factors together with blood biomarkers in the relation to the amount of ctHPV16 at presentation.

## Material and methods

### Patient characteristics

Patients admitted to National Research Institute of Oncology, Gliwice Branch, Poland due to HPV + OPC between 2012 and 2022 were retrospectively analysed. The group consisted of 55 patients with HPV + OPC with ctHPV16 assessed at diagnosis and computerized tomography (CT), magnetic resonance imaging (MRI) or 18F-fluorodeoxyglucose positron emission tomography (PET-CT) scan acquisition prior to treatment. Four patients were excluded from analysis due to missing data, leaving a sample size of 51. There were 37 men and 14 women with a median age of 57 years (IQR: 49–63). Stage of primary tumor (T) and involved lymph nodes (N) were assessed according to AJCC8. Primary symptoms of disease were based on clinical interview with the patient via the medial record.

Blood derived factors included squamous cell carcinoma antigen (SCC-Ag), serum soluble fragment of cytokeratin 19 (CYFRA 21-1), C-reactive protein (CRP), albumin level (Alb), neutrophils (Neut), thrombocytes (Plt) and lymphocyte (Lym) count, Neut/Lym ratio based on routine clinical results were assessed. The concentration of SCC-Ag was determined by chemiluminescent microparticle immunoassay (CMIA), using analyzer Alinity I and commercial kit analyzer from Abbott Laboratories (Abbott Park, IL, USA). The concentration of CYFRA 21-1 was determined by means of the electrochemiluminescence immunoassay method (ECLIA) using the Roche Diagnostics (Basel, Switzerland) reagent kits and Cobas e801 analyzer. Serum CRP and albumin concentrations were measured by immunonephelometric technique, using a Siemens reagent kit and a Atelica nephelometer. The lymphocyte-to-monocyte ratio was calculated using automated complete blood cell count (CBC) data obtained by dividing the lymphocyte-to-monocyte count. A Sysmex XN 2000 analyzer machine was used for automated CBC.

For descriptive statistics, see Table S1 and S2 in the supplementary materials. Written informed consent was required from all participating patients.

Informed consent was taken from all participants. Institutional Review Board Statement: The study was conducted according to the guidelines of the Declaration of Helsinki, and approved by the Bioethics Committee at Maria Sklodowska‑Curie National Research Institute of Oncology Gliwice Branch (protocol KB/430‑24/19 date of approval 11 March 2019).

### Detection of HPV16 in plasma

Peripheral blood (12 mL) was collected in K3EDTA tubes (Becton–Dickinson, Franklin Lakes, NJ, USA). Plasma was separated within an hour by double centrifugation at 300 × g and 1000 × g, both at 4 °C for 10 min. DNA was extracted (according to the manufacturer’s instructions) from 1 mL of plasma using the Genomic Mini AX Body Fluids Kit (A&A Biotechnology, Gdynia, Poland). PCR reactions were performed using the Bio-Rad CFX96 qPCR instrument (Bio-Rad Laboratories, Hemel Hempstead, UK). Each measurement consisted of a standard curve of three dilutions of a plasmid construct containing the HPV16 and TERT genome, a negative control, and a sample. The amplification of TERT (human telomerase reverse transcriptase) was used for measurement of the total cell-free DNA in blood. The obtained copies of ctHPV16 were calculated according to the amount of plasma that was taken for DNA extraction (copies/ml) − viral load (VL). ctHPV16 VL in plasma was expressed as a log10 of copy number of HPV16 DNA per 1 mL (log10 viral load, log10 VL). After conversion of the values to log10, the range between 0.4 and 5.9 were obtained with a normal distribution. The measured ctHPV16 as a viral load (VL) of HPV16-DNA in plasma was expressed as a log10 of copy number of HPV16 DNA/ml.

### The assessment of the tumor volume

The volume of primary tumor (TV) and involved lymph nodes (NV) of the neck were calculated independently. MRI examinations were performed with a 1.5 T and 3 T field induction apparatus. TV and NV were calculated in T1-w DCE (T1-weighted dynamic contrast enhancement) after intravenous administration of a paramagnetic contrast agent.

PET-CT examinations were performed after intravenous administration of 18F-Fluorodeoxyglucose (18F FDG). The CT examination was performed using the spiral technique with the reconstruction of 3 mm layers without providing contrast iv. The metabolic tumor volume (MTV) of the lesions was counted at 30%, 40% and 50% of the maximum normalized uptake (SUV) and additionally with an SUV cut-off threshold of 2.5.

Computed tomography (CT) volumes were calculated on contrast-enhanced images. The areas of the primary tumor and lymph nodes with a metastatic image were segmented after contrast agent administration.

### Statistical analysis

Categorical variables were summarized as frequencies and percentages. Continuous data were shown as median values with interquartile ranges (25% to 75%, IQR 25–75) and mean values with standard deviation/min/max ranges, unless otherwise stated. For exact descriptive statistics, see Table S1 and S2 in the supplementary materials.

Predictive mean matching was used to replace missing data with use of mice package (v. 3.15.0) ^23^. Table S3 in supplementary materials represents the amount of missing data per patient that was included or excluded from the study.

Data were analysed using parametric and nonparametric methods depending on distribution and homogeneity of variance. Normality of distribution was tested using the Shapiro–Wilk test. To evaluate the difference in VL level the one-way analysis of variance (ANOVA with Duncan’s adjustment for pairwise comparisons) and Student's t-test was performed with d Cohen effect size calculation. The correlation with VL was investigated using stats package (v. 3.6.2) ^24^ (Pearson’s, Spearman's rank or Point biserial correlation was applied accordingly).

Visualizations was prepared with the ggplot2 (v. 3.4.0) ^25^ and corrplot package (v. 0.92) ^26^.

The following variables were assessed with VL as continuous and as categorical variables: TV, NV, combined TV and NV (TV + NV), SCC, age, symptoms duration (time to diagnosis − TtDGN) and CYFRA 21-1. Categorization was made according to median value.

In order to assess predictor(s) of VL level a univariable and multivariable linear regression analysis was performed with reduction in a stepwise manner. Models were compared on the basis of AIC (Akaike's Information Criterion). Analysis were performed using AICcmodavg (v. 2.3-1) ^27^, tidyverse (v. 1.3.0) ^25,28^, ggpubr (v. 0.5.0) ^29^, and MASS package (v. 7.3-58.1) ^30^.

Variables for multivariable analysis were chosen based on the results from univariable analysis (correlation, regression and difference). Full report can be found in Table S6 in Supplementary Material.

All analyses were performed using the R environment for statistical computing version 4.1.3 "One Push-Up" released on 10 March 2022 (R Foundation for Statistical Computing, Vienna, Austria, http://www.r-project.org). A two-sided P value < 0.05 was considered statistically significant, and a P value < 0.10 was considered close statistical significance. The Benjamini–Hochberg correction was applied to account for multiple testing, where a q-value of < 0.05 was considered statistically significant. Since the correction did not alter the inference, q-values are reported accordingly in the Supplementary Materials.

## Results

Nine (18%), 10 (19%) and 32 (63%) patients had TV and NV assessed by MRI, CT or PET respectively.

In univariable analysis there was no difference in ctHPV16 level by median age, sex, disease symptoms or smoking status (Table 1; Supplementary materials, Table S4). However, pain was associated with ctHPV16 level (P = 0.038, d = 0.613). Primary tumor neither as T-stage nor TV (Table 1 and S4) was related to ctHPV16. On the contrary, involved regional nodes were related to VL with a significant difference in VL for N1 patients vs N3 patients (P = 0.044, d = 0.857; Table 1).

**Table 1:** The difference in the mean value of initial ctHPV16 VL for various clinical factors.

Variable	Category	Total number of patients (N = 51)	ctHPV16 Mean (SD)	P value*		Cohen's d
Sex	Males	37	3.410 (0.982)	0.186		–
	Females	14	2.935 (1.380)			
Age	≤ 57	26	3.237 (0.406)	0.787		–
	> 57	25	3.324 (1.046)			
Smokers	Yes	22	3.316 (1.096)	0.845		–
	No	29	3.252 (1.147)			
Symptoms	Pain Yes	24	2.932 (1.012)	**0.038**		**0.613**
	Pain No	27	3.589 (1.132)			
	Neck tumor Yes	31	3.233 (1.075)	0.720		–
	Neck tumor No	20	3.352 (1.197)			
	Weight loss Yes	9	2.892 (0.827)	0.263		–
	Weight loss No	42	3.363 (1.163)			
	1 symptom	27	3.498 (1.056)	0.147		–
	> 1 symptom	24	3.034 (1.151)			
	2 symptoms	14	3.166 (1.265)	0.666		–
	> 2 symptoms	37	3.323 (1.065)			
	> 2 symptoms	7	2.940 (0.790)	0.401		–
	≤ 2 symptoms	44	3.334 (1.161)			
Symptoms duration (months)	≤ 5	27	3.086 (1.073)	0.201		–
	> 5	24	3.497 (1.144)			
SCC-Ag (median)	≤ 1.9	29	3.286 (1.117)	0.967		–
	> 1.9	22	3.272 (1.136)			
CYFRA 21-1 (median)	CYFRA 21-1 low ≤ 3.75	26	2.820 (1.042)	**0.002**		**0.917**
	CYFRA 21-1 high > 3.75	25	3.758 (1.003)			
T	1	5	3.482 (1.053)	0.884		–
	2	22	3.364 (0.948)			
	3	16	3.221 (1.158)			
	4	8	3.039 (1.457)			
N	1	14	2.774 (0.954)	1 vs. 2: 0.301	**0.076**	–
	2	13	3.184 (1.229)	1 vs. 3:** 0.044**		**0.857**
	3	24	3.626 (1.035)	2 vs. 3: 0.264		–
TV (cm^3^)	TV low ≤ 32.45	26	3.082 (1.095)	0.208		–
	TV high > 32.45	25	3.486 (1.121)			
NV (cm^3^)	NV low ≤ 26.92	26	2.914 (1.122)	**0.023**		**0.703**
	NV high > 26.92	25	3.660 (0.996)			
TV + NV (cm^3^)	TV + NV low ≤ 60.54	26	2.931 (0.988)	**0.018**		**0.827**
	TV + NV high > 60.54	25	3.642 (1.145)			
CRP (median)	CRP low ≤ 2.03	27	2.933 (1.096)	**0.019**		**0.694**
	CRP high > 2.03	24	3.670 (1.027)			
Alb (median)	Alb low ≤ 43	26	3.091 (1.293)	0.231		
	Alb high > 43	25	3.476 (0.878)			
Neut/Lym (median)	Neut/Lym low ≤ 2.25	26	3.179 (1.064)	0.526		
	Neut/Lym high > 2.25	25	3.384 (1.178)			
Neut (median)	Neut low ≤ 4.3	26	3.159 (1.149)	0.445		
	Neut high > 4.3	25	3.405 (1.087)			
Limf (median)	Limf low ≤ 1.89	26	3.528 (1.221)	0.113		
	Limf high > 1.89	25	3.022 (0.951)			
WBC (median)	WBC low ≤ 6.99	26	3.416 (1.260)	0.387		
	WBC high > 6.99	25	3.138 (0.945)			
Plt (median)	Plt low ≤ 259 000	26	3.340 (1.158)	0.702		
	Plt high > 259 000	25	3.217 (1.088)			

Significant values are in bold.

*The Benjamini–Hochberg correction (q-value) can be found in the Supplementary Materials—Table S10.

**Figure 1:**
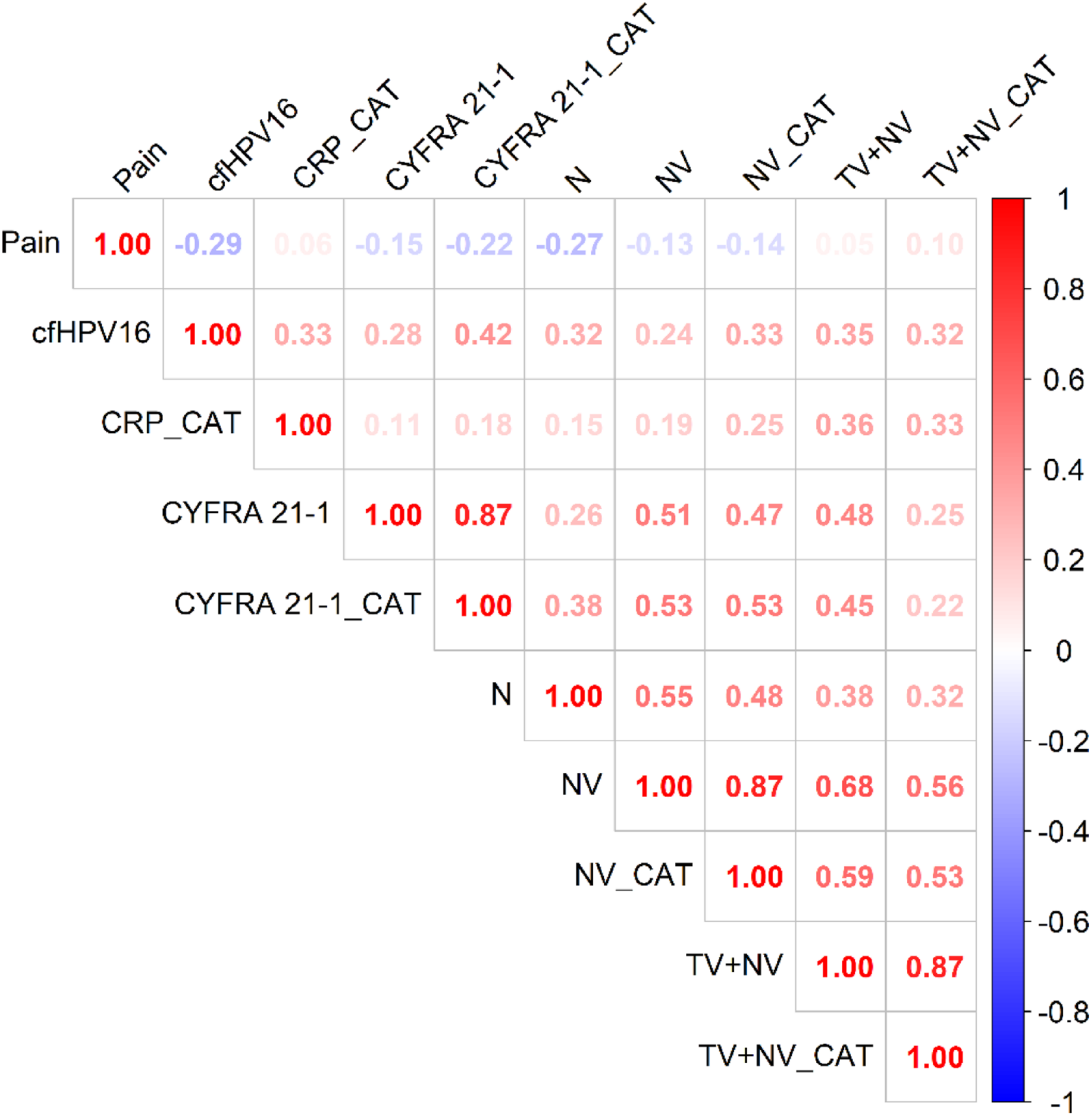
Graphical display of a significant correlations between cfHPV16 VL and analyzed variables.

To assess how selected tumor- and patient-related factors together with blood biomarkers affect ctHPV16, a correlation analysis was performed. VL was significantly correlated with CYFRA 21-1 median (both as continues and as categorical variable: rho = 0.331, P = 0.017 and r = 0.417, P = 0.002 accordingly), N stage (rho = 0.386, P = 0.005), NV (both as continues and as categorical variable: rho = 0.360, P = 0.009 and r = 0.331, P = 0.018 accordingly), TV + NV (both as continues and as categorical variable: rho = 0.424, P = 0.002 and r = 0.316, P = 0.024), CRP level as categorical variable (r = 0.327, P = 0.019), and pain (r =  − 0.291, P = 0.038) (Fig. 1). The complete report on correlation between VL and analysed variables is given in supplementary materials (Table S4; Fig. S1).

Next, variables were divided according to median values and related to VL.

**Figure 2:**
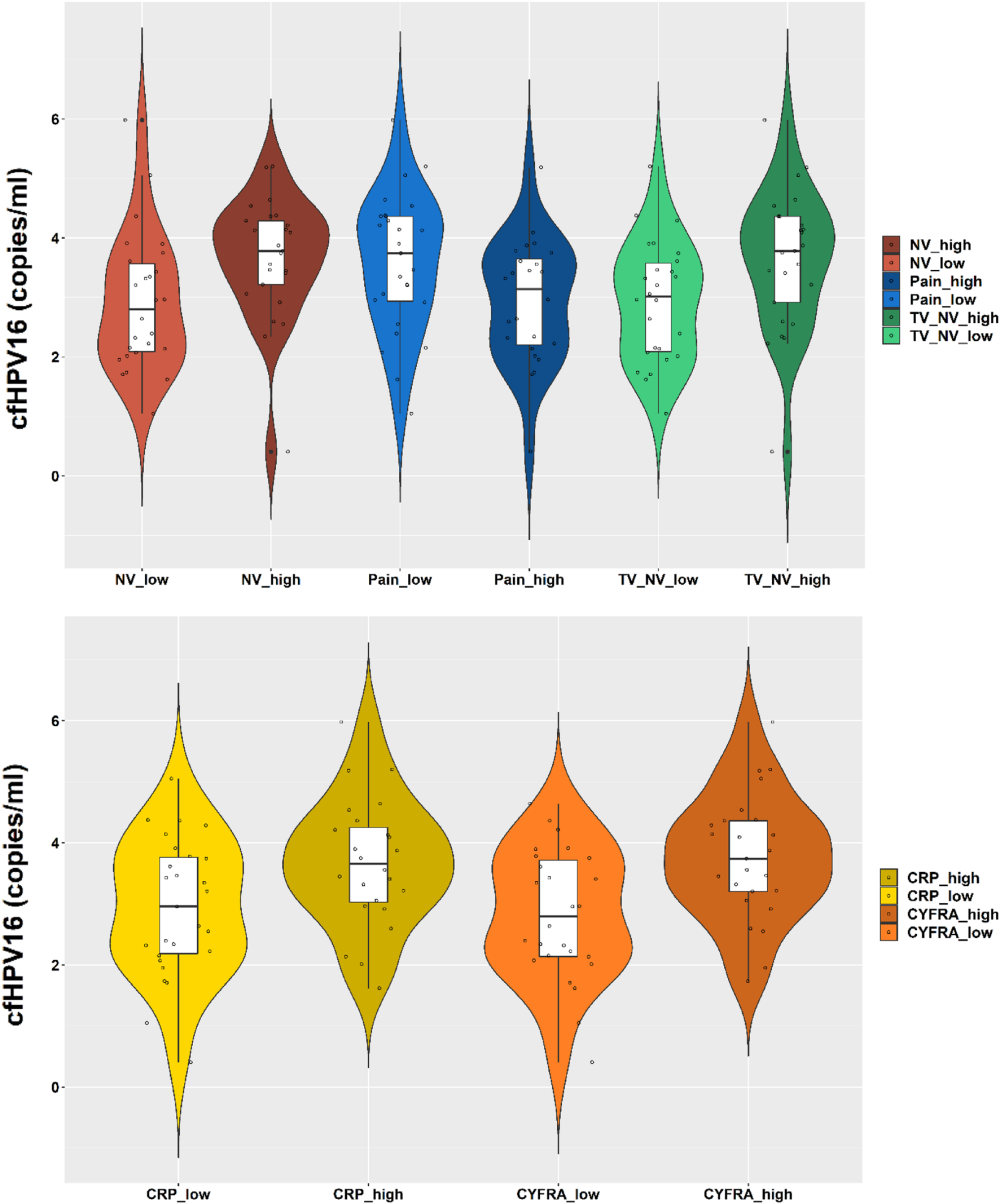
Violin plots with box plots presenting difference in cfHPV16 VL between patients with larger and smaller volumes of nodes or TV + NV, or between patients with smaller value and a larger value of CYFRA 21-1, CRP and pain.

Significant difference in VL was observed between patients with median/below median and above median of NV (P = 0.023, d = 0.703), TV + NV (P = 0.018, d = 0.827), CYFRA 21-1 (P = 0.002, d = 0.917) and CRP (P = 0.019, d = 0.694). Significant difference in VL was also observed, between patients with and without pain (P = 0.038, d = 0.613) (Fig. 2; Table 1).

CYFRA 21-1 and TV + NV both, as continuous and as categorical variable, N, pain, NV, and CRP as a categorical variable and TV as continues variable appeared to be a significant predictors of VL level (Table S5 in the Supplementary Material).

For multivariable analysis variables: pain, N, NV_CAT (NV as a categorical variable), CYFRA_CAT, and CRP_CAT (CYFRA 21-1 and CRP as a categorical variable accordingly) were divided into groups. Group I consisted of variables related to tumor and patient (pain, N, NV_CAT) and Group II consisted of variables related to biochemical biomarkers (CYFRA_CAT, CRP_CAT). From the group I NV as categorical variable (Table 2; Table S7) and all of the variables from the group II came as significant predictors of ctHPV16 level (Table 3; Table S8). Full assessment report can be found in Table S6.

**Table 2:** Multivariable linear regression analysis with predictors related to tumor condition.

Full model				Reduced model			
Variable	Estimate	95% CI	P value	Variable	Estimate	95% CI	P value**
N	0.205	− 0.208 to 0.617	0.324				
NV_CAT	0.515	− 0.160 to 1.190	0.132	NV_CAT	0.667	0.067 to 1.268	0.030*
Pain	− 0.494	− 1.112 to 0.125	0.115	Pain	− 0.564	− 1.166 to 0.038	0.065

Significant values are in bold.

*NV_CAT—nodal volume categorised below and over median value, N—N-stage acc to AJCC 8th. **The Benjamini–Hochberg correction (q-value) can be found in the Supplementary Materials—Table S11.

**Table 3:** Multivariable linear regression analysis with predictors related to biochemical factors.

Full model				Reduced model**			
Variable	Estimate	95% CI	P value	Variable	Estimate	95% CI	P value***
CYFRA_CAT	0.835	0.256–1.414	**0.006**	CYFRA_CAT	0.835	0.256–1.414	0.006*
CRP_CAT	0.590	0.0108–1.170	**0.046**	CRP_CAT	0.590	0.0108–1.170	0.046*

Significant values are in bold.

*CYFRA_CAT—level of CYFRA 21-1 categorised below and over median value, CRP_CAT—C-Reactive Protein concentration categorised below and over median value. **Full model could not be reduced further to get a better model. ***The Benjamini–Hochberg correction (q-value) can be found in the Supplementary Materials—Table S12.

## Discussion

The detection of HPV sequences in the total cell free DNA in the blood of patients with HPV + OPC has become the basis of ctHPV assays as a tumor-derived biomarker ^31–33^. The amount of viral DNA could be described as a VL which is the number of viral particles per millilitre of the blood. Importantly, ctHPV levels, do not always correlate with the clinical stage of the cancer. For patients with HPV + OPC, VL is strongly, positively correlated with tissue VL − tVL (tVL) and the probability of detecting ctHPV in the blood increases with the increase of tVL ^10^. Cao et al. reported that the low tVL could be one of the contributors influencing the inability to detect ctDNA in the blood at baseline ^20^. Chera et al. found that patients with low baseline ctHPV (</= 200 copies/mL) in the blood had a significantly lower tVL than patients with high baseline ctHPV (> 200 copies/mL) ^13^. Understanding what clinical factors may influence ctHPV levels is critical for understanding the role of ctHPV in prognostication and treatment monitoring.

Certain populations of cancer cells may produce various amount of ctHPV. Moreover, distinct mechanisms influence ctDNA release which can be early or delayed. The kinetics of releasing ctHPV depends not only on the type of cancer cell but also external cytotoxic factors. Further, cell death mechanisms seem to be relevant as well. Cellular senescence contrary to immediate apoptotic death or necrosis may change the kinetics of ctDNA delaying ctDNA release ^12^. Furthermore, the structure of tumor including vascularity, perfusion, or hypoxia may also be confounding factors affecting the shedding rate of ctDNA ^34^.

A common features of HPV + OPC is a small primary tumor and higher volume nodal involvement, compared to non-HPV-associated OPC, and nearly all patients with HPV + OPC present with lymph node metastases ^2,35–37^. Thus, the questions remains what are the primary factors driving ctHPV.

While analysing primary tumor as a T-stage we did not find any correlation with ctHPV. This is in concordance with other reports. Chera et al. found even that patients with T3-T4 tumor stage of HPV + OPC had significantly lower baseline levels of ctHPV than patients with T2 tumor, suggesting that larger tumor size may be associated with lower rates of ctHPV release ^13^. Further analysis revealed that the level of baseline ctHPV may relate to HPV integration within the host genome, with more integrated cases having lower baseline ctHPV. Dahlstrom et al. reported no relationship between pretreatment serum ctHPV level and T-stage ^38^. In another study Cao et al. found no correlation between T-stage and ctHPV even if metabolic volume of primary tumor was taken into account ^20^. The lack of correlation between T-stage and ctHPV may be also the result of T-classification that reflects more the area of cancer infiltration and involvement of surrounding structures than the volume of the tumor. This difference between dimension and volume increases in cubic relation and is more meaningful for larger tumors. Also for patients with cervical cancer the relation between stage of disease and ctHPV16 was not obvious with significant difference between stage I and II but not between II and III or II and IV stage ^7^.

Contrary to primary tumor, we found significantly lower ctHPV levels in N1 tumors vs N3. Between stage N1 and N2 the difference was not significant. This discrepancy may be explained by staging features in AJCC 8th. N2 staging involves patients with metastatic lymph nodes on contralateral or both sides of the neck, independent of its dimension. Although 8th staging edition tried to better stratify HPV + OPC, it is not focused on tumor quantity. Due to this, besides T and N classification we also radiologically estimated the TV and NV what may more accurately reflect the quantify of tumor. For combined TV and NV, the median volume over 56.64 cm^3^ reflected significantly higher level of ctHPV. As previously mentioned, Dahlstrom et al. found that pretreatment ctHPV increased with increasing N category and was significantly higher while accounting stage of disease (combined T and N category) ^38^. Retting et al. considered diameter of primary tumor and involved nodes according to dimensions based on standard contrast-enhanced CT. They reported strong association of N stage with ctHPV while only a weak association with clinical T stage. Notably, in this set of patients T0 presented the highest ctHPV. Also the size of the largest cervical lymph node presented a stronger association with ctHPV than size of the primary tumor ^22^. Results of functional imaging also indicated that ctHPV is related more to NV than to TV. Cao et al. also found that pretreatment ctHPV was significantly correlated with NV, nodal poorly perfused subvolumes and high cellular subvolume of involved nodes defined from MRI. Authors suggested that cellularly dense nodal burden may dominate in the releasing of tumor DNA ^20^. Retting et al. reported only moderated association between metabolic activity in both primary tumor, nodes and ctHPV, although increasing SUVmax in involved lymph nodes was related to progressive increase of ctHPV ^22^.

Additional adverse clinical risk factor as smoking also may influence the relation between ctHPV and tumor burden. Chera et al. found that patients with more than 10 tobacco package per year had lower baseline ctHPV level despite T4 tumor stage ^13^. Hashida et al. reported that patients with ≥ 20 pack-years had significantly lower ctHPV ^39^. Mazurek et al. found that particularly high tVL was found in non-smoking women ^10^. This may suggest that in patients with HPV + OPC smoking, as an additional risk factor is responsible more for advanced disease rather than HPV and that prognosis based on pretreatment ctHPV in such patients may be of questionable value.

Significant correlation between ctHPV and levels of CYFRA 21-1 and CRP in the blood was found in our patients. CYFRA 21-1 is the serum soluble fragment of cytokeratin 19 and is a part of cytoskeleton of epithelial cell. CYFRA 21-1 is released by epithelium-derived cancer cells while proliferating. In some reports the correlation between CYFRA 21-1 and tumor growth, stage of disease, involved lymph nodes ^40^ or distant metastases ^41^ has been found. However, an inverse correlation between CYFRA 21-1 and the grade of tumor has been also reported ^42^. Little is known about CYFRA 21-1 in patients with HPV + OPC. Rudhart et al. did not find significant differences of the CYFRA 21-1 serum concentration for patients with HPV-associated and not associated tumors prior to treatment; however over 40% of patients in this group had unknown HPV status ^43^. Despite some uncertainties of CYFRA 21-1 as a marker of active tumor, we found that the median value of CYFRA 21-1 significantly separated patients in our group according to ctHPV, suggesting that CYFRA 21-1 may reflect higher proliferation of tumor cells.

Serum C-reactive protein (CRP) is a marker of inflammation and is elevated in response to tissue damage or infection. CRP level is also correlated with both tumor and nodal stage and histopatological differentiation of the tumor. Our results showed that patients with median CRP value over 2.03 mg/l had significantly higher ctHPV level. Husuan Ho et al. found that in the group of patients with pharyngolaryngeal carcinoma CRP level was significantly related to maximal SUV of involved nodes but not primary tumor ^44^. Johnson-Obaseki et al. ^45^ reported that CRP level was significantly higher in the patients with HPV + OPC but contrary results were described by Xiao et al. ^46^. The elevation of CRP also arises from the host immune responses to tumor growth with elevated inflammatory cytokines, especially Il-6 ^44^. Our results confirm that ctHPV is significantly related to the level of both CYFRA 21-1 and CRP. The clinical utility of the assessment of these markers in patients with HPV + OPC and its potential prognostic or predictive value require however further investigation.

HPV DNA can be found as both, integrated and episomal states in cancer cells. Traditionally, episomal HPV has been felt to correlate with a favourable prognosis while integrated tumors were felt to have adverse tumor genomic feature ^39,47^. Lower initial level of ctHPV DNA has been reported to be associated with clinically higher-risk disease and also with greater likelihood of HPV integration, which represents adverse tumor genomic features ^13^. Recently, a more complex understanding of HPV genome states has emerged. Rossi et al. showed novel mechanism for HPV16 to cause cancer without integration through aberrant episomal replication, forming rearranged, mutated, and multimer episomes ^48^.

Finally, it should be noticed that in patients with no pain as clinical symptom of cancer, ctHPV was significantly higher. Explanation of this remain to be elucidated especially due to the assumption that the level of ctHPV seems to be related to lymphovascular invasion ^22^ and invasive component usually is responsible for the pain. This effect however might be less pronounced for HPV-associated primaries less likely to display ulceration or necrosis or nodal disease that is generally more clustered and cystic than for HPV-not associated tumors usually presenting ill-defined borders and invading into neighbouring muscle ^49^. There is also the general observation that patients with HPV-associated tumor usually are in better general performance status being less fatigue and less devastated by cancer disease that their counterparts with HPV-not associated tumors ^46^.

This study has several limitations. The study is retrospective, which may lead to data inaccuracies. Other doubts may be related to three distinct methods of tumor volume assessment. Despite of this, to our knowledge, the present study is the first to investigate correlation between volume of the primary tumor, lymph nodes and ctHPV. The significant correlation between tumor markers like CYFRA 21-1 or CRP and ctHPV has also not been previously described.

## Conclusions

Our results indicate that ctHPV16 at presentation is driven by tumor volume measured mostly by N what is in concordance with other data suggesting nodal disease as the main determinant of this marker. CYFRA 21-1 and CRP are additional factors related to ctHPV16 prior to the treatment. This data contribute to the growing body of literature leading to a better understanding of the factors that influence ctHPV levels at diagnosis, which could help identify distinct subpopulations of patients with HPV + OPC for prognostication and stratification.

### Supplementary Information

Supplementary Information.

## Data Availability

The datasets used and analyzed during the current study are available from the corresponding author on reasonable request.
