# Supplementary material for: Determinants of the level of circulating-tumor HPV16 DNA in patients with HPV-associated oropharyngeal cancer at the time of diagnosis
Source: Sci Rep. 2023 Dec 1;13:21226. doi: 10.1038/s41598-023-48506-6 (PMC10692143; doi:10.1038/s41598-023-48506-6)
Supplement: Supplementary file 1 — Supplementary Information. [file 41598_2023_48506_MOESM1_ESM.docx]

**Supplementary Material**

**Table S1.** Descriptive statistics - qualitative variables.

| **Variable** | **Category** | **Total number**  **of patients (N = 51)** | **%** |
| --- | --- | --- | --- |
| sex | Males  Females | 37  14 | 72.549  27.451 |
| Smokers | Yes  No | 22  29 | 43.137  56.863 |
| Symptoms | Pain Yes  Pain No | 24  27 | 47.059  52.941 |
|  | Neck tumour Yes  Neck tumour No | 31  20 | 60.784  39.216 |
|  | Weigh loss Yes  Weigh loss No | 9  42 | 17.647  82.353 |
|  | 1 symptom  > 1 symptom | 27  24 | 52.941  47.059 |
|  | 2 symptoms  1 & > 2 symptoms | 14  37 | 27.451  72.549 |
|  | > 2 symptoms  <= 2 symptoms | 7  44 | 13.725  86.275 |
| T | 1  2  3  4 | 5  22  16  8 | 9.804  43.137  31.373  15.686 |
| N | 1  2  3 | 14  13  24 | 27.451  25.490  47.059 |
|  |  |  |  |
|  |  |  |  |

**Table S2.** Descriptive statistics – continuous variables.

| **No** | **Variable** | **N** | **Mean** | **SD** | **min** | **Q1** | **Median** | **Q3** | **max** |
| --- | --- | --- | --- | --- | --- | --- | --- | --- | --- |
| 1. | Age | 51 | 56 | 10 | 30 | 49 | 57 | 63 | 73 |
| 2. | TtDGN (months) | 51 | 6.647 | 7.506 | 0 | 3 | 5 | 8 | 44 |
| 3. | TV (cm3) | 51 | 34.881 | 26.062 | 2.700 | 14.755 | 32.457 | 46.200 | 124.840 |
| 4. | NV (cm3) | 51 | 34.656 | 28.397 | 0.470 | 10.395 | 26.920 | 47.275 | 108.272 |
| 5. | TV+NV (cm3) | 51 | 69.536 | 42.188 | 12.260 | 42.350 | 60.540 | 84.300 | 195.430 |
| 6. | cfHPV16 (copies/ml) | 51 | 3.280 | 1.126 | 0.406 | 2.366 | 3.405 | 4.109 | 5.979 |
| 7. | SCC-Ag | 51 | 6.733 | 10.755 | 0.300 | 1.300 | 1.900 | 5.700 | 49.300 |
| 8. | CYFRA 21-1 | 51 | 5.906 | 5.353 | 1.030 | 2.905 | 3.750 | 6.325 | 27.420 |
| 9. | Alb | 51 | 43.392 | 3.986 | 28.000 | 42.000 | 43.000 | 46.000 | 50.000 |
| 10. | CRP | 51 | 5.340 | 7.506 | 0.205 | 0.928 | 2.030 | 5.885 | 30.100 |
| 11. | Limf | 51 | 1.889 | 0.602 | 0.770 | 1.470 | 1.890 | 2.145 | 3.620 |
| 12. | Neut | 51 | 4.731 | 1.779 | 2.230 | 3.620 | 4.300 | 5.305 | 10.980 |
| 13. | Plt | 51 | 272.353 | 67.670 | 156.000 | 224.500 | 259.000 | 316.500 | 436.000 |
| 14. | WBC | 51 | 7.417 | 2.031 | 3.080 | 6.205 | 6.990 | 7.765 | 14.540 |
| 15. | Neut/Lym | 51 | 2.822 | 1.552 | 1.116 | 1.756 | 2.252 | 3.513 | 7.522 |

**Table S3.** Missing data per patient included and excluded from the study.

| **Patient Number** | **Missing data**  **n** | **Missing data**  **%** |
| --- | --- | --- |
| 23 | 9 | 34.62 |
| 24 | 9 | 34.62 |
| 25 | 9 | 34.62 |
| 26 | 9 | 34.62 |
| 5 | 4 | 15.38 |
| 9 | 4 | 15.38 |
| 10 | 4 | 15.38 |
| 18 | 4 | 15.38 |
| 41 | 4 | 15.38 |
| 54 | 4 | 15.38 |
| 2 | 2 | 7.69 |
| 8 | 2 | 7.69 |
| 36 | 2 | 7.69 |
| 47 | 2 | 7.69 |
| 49 | 2 | 7.69 |
| 50 | 2 | 7.69 |
| 55 | 2 | 7.69 |
| 7 | 1 | 3.85 |
| 11 | 1 | 3.85 |
| 19 | 1 | 3.85 |
| 43 | 1 | 3.85 |
| 48 | 1 | 3.85 |
| 1 | 0 | 0.00 |
| 3 | 0 | 0.00 |
| 4 | 0 | 0.00 |
| 6 | 0 | 0.00 |
| 12 | 0 | 0.00 |
| 13 | 0 | 0.00 |
| 14 | 0 | 0.00 |
| 15 | 0 | 0.00 |
| 16 | 0 | 0.00 |
| 17 | 0 | 0.00 |
| 20 | 0 | 0.00 |
| 21 | 0 | 0.00 |
| 22 | 0 | 0.00 |
| 27 | 0 | 0.00 |
| 28 | 0 | 0.00 |
| 29 | 0 | 0.00 |
| 30 | 0 | 0.00 |
| 31 | 0 | 0.00 |
| 32 | 0 | 0.00 |
| 33 | 0 | 0.00 |
| 34 | 0 | 0.00 |
| 35 | 0 | 0.00 |
| 37 | 0 | 0.00 |
| 38 | 0 | 0.00 |
| 39 | 0 | 0.00 |
| 40 | 0 | 0.00 |
| 42 | 0 | 0.00 |
| 44 | 0 | 0.00 |
| 45 | 0 | 0.00 |
| 46 | 0 | 0.00 |
| 51 | 0 | 0.00 |
| 52 | 0 | 0.00 |
| 53 | 0 | 0.00 |

**Table S4.** Correlation between ctHPV16 and analyzed variables.

| **No** | **Variable** | **rho/r** | **95% CI** | **P value** | **q-value** | **Test** |
| --- | --- | --- | --- | --- | --- | --- |
| 1. | TV+NV | **0.424** | 0.168 - 0.626 | **0.002** | **0.004** | Spearman's rank correlation |
| 2. | CYFRA 21-1_CAT | **0.417** | 0.160 - 0.621 | **0.002** | **0.006** | Point Biserial Correlation |
| 3. | N | **0.386** | 0.123 - 0.598 | **0.005** | **0.009** | Spearman's rank correlation |
| 4. | NV | **0.360** | 0.093 - 0.578 | **0.009** | **0.013** | Spearman's rank correlation |
| 5. | CYFRA 21-1 | **0.331** | 0.061 - 0.556 | **0.017** | **0.017** | Spearman's rank correlation |
| 6. | NV_CAT | **0.331** | 0.061 - 0.556 | **0.018** | **0.020** | Point Biserial Correlation |
| 7. | CRP_CAT | **0.327** | 0.056 - 0.553 | **0.019** | **0.026** | Point Biserial Correlation |
| 8. | TV+NV_CAT | **0.316** | 0.044 - 0.544 | **0.024** | **0.033** | Point Biserial Correlation |
| 9. | Pain | **-0.291** | -0.525 - -0.017 | **0.038** | **0.042** | Point Biserial Correlation |
| 10. | Limf_CAT | -0.225 | -0.471 - 0.054 | 0.113 | 0.061 | Point Biserial Correlation |
| 11. | 1 symptom | -0.206 | -0.456 - 0.074 | 0.147 | 0.066 | Point Biserial Correlation |
| 12. | CRP | 0.189 | -0.092 - 0.441 | 0.181 | 0.070 | Spearman's rank correlation |
| 13. | TtDGN | 0.187 | -0.093 - 0.440 | 0.184 | 0.072 | Pearson correlation |
| 14. | Sex | -0.188 | -0.441 - 0.092 | 0.186 | 0.075 | Point Biserial Correlation |
| 15. | TV | 0.186 | -0.095 - 0.439 | 0.187 | 0.079 | Spearman's rank correlation |
| 16. | TtDGN_CAT | 0.182 | -0.099 - 0.436 | 0.201 | 0.083 | Point Biserial Correlation |
| 17. | TV_CAT | 0.180 | -0.101 - 0.434 | 0.208 | 0.088 | Point Biserial Correlation |
| 18. | Alb | 0.174 | -0.107 - 0.429 | 0.218 | 0.094 | Spearman's rank correlation |
| 19. | Alb_CAT | 0.171 | -0.110 - 0.426 | 0.231 | 0.097 | Point Biserial Correlation |
| 20. | Weigh loss | -0.160 | -0.417 - 0.121 | 0.263 | 0.103 | Point Biserial Correlation |
| 21. | Age | 0.127 | -0.154 - 0.389 | 0.374 | 0.110 | Pearson correlation |
| 22. | Age  WBC_CAT | 0.142 | -0.139 - 0.402 | 0.314 | 0.112 | Spearman's rank correlation |
|  |  | -0.124 | -0.386 - 0.157 | 0.387 | 0.117 | Point Biserial Correlation |
| 23. | > 2 symptoms | 0.120 | -0.161 - 0.383 | 0.401 | 0.123 | Point Biserial Correlation |
| 24. | Plt | -0.116 | -0.380 - 0.165 | 0.412 | 0.127 | Spearman's rank correlation |
| 25. | T | -0.115 | -0.379 - 0.165 | 0.415 | 0.128 | Spearman's rank correlation |
| 26. | Neut_CAT | 0.109 | -0.171 - 0.374 | 0.445 | 0.134 | Point Biserial Correlation |
| 27. | Neut/Lym_CAT | 0.091 | -0.189 - 0.358 | 0.526 | 0.139 | Point Biserial Correlation |
| 28. | WBC | -0.084 | -0.352 - 0.196 | 0.552 | 0.143 | Spearman's rank correlation |
| 29. | 2 symptoms | 0.062 | -0.217 - 0.332 | 0.666 | 0.145 | Point Biserial Correlation |
| 30. | Limf | -0.062 | -0.332 - 0.218 | 0.667 | 0.150 | Pearson correlation |
| 31. | Limf  Plt_CAT | -0.089 | -0.356 - 0.191 | 0.529 | 0.154 | Spearman's rank correlation |
|  |  | -0.055 | -0.326 - 0.224 | 0.702 | 0.160 | Point Biserial Correlation |
| 32. | Neck tumour | -0.051 | -0.322 - 0.227 | 0.720 | 0.167 | Point Biserial Correlation |
| 33. | Age_CAT | 0.039 | -0.239 - 0.311 | 0.787 | 0.172 | Point Biserial Correlation |
| 34. | Smoking | 0.028 | -0.250 - 0.301 | 0.845 | 0.178 | Point Biserial Correlation |
| 35. | Neut | -0.026 | -0.299 - 0.252 | 0.857 | 0.183 | Spearman's rank correlation |
| 36. | Neut/Lym | 0.025 | -0.253 - 0.298 | 0.862 | 0.185 | Spearman's rank correlation |
| 37. | SCC-Ag | -0.023 | -0.297 - 0.254 | 0.871 | 0.187 | Spearman's rank correlation |
| 38. | SCC-Ag_CAT | -0.006 | -0.281 - -0.270 | 0.967 | 0.196 | Point Biserial Correlation |


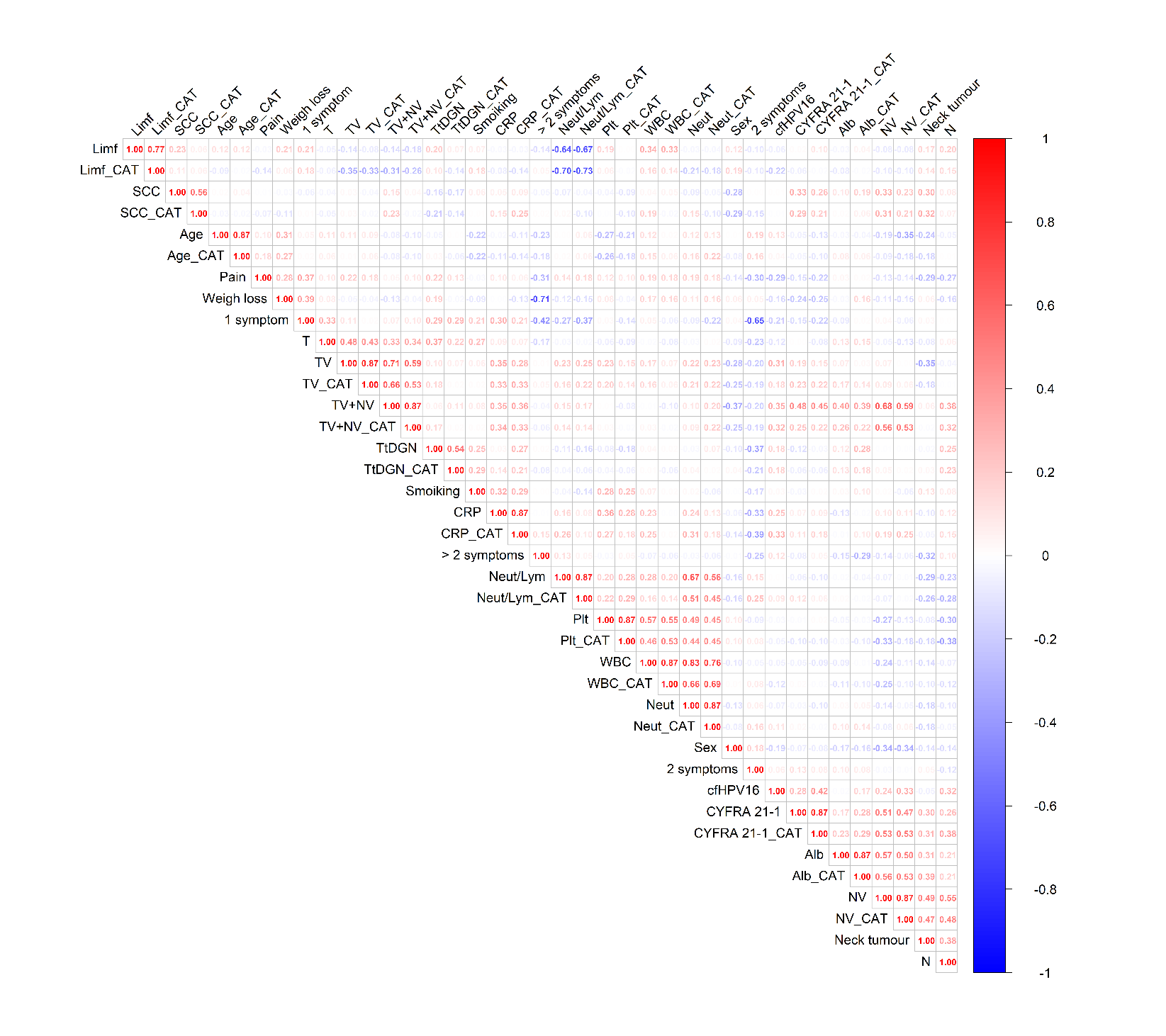


**Fig. S1.** Graphical display of a correlation matrix of all analyzed variables.

**Table S5.** Univariable linear regression analysis.

| **No** | **Variable** | **Estimate** | **95% CI** | **AIC** | **P value** | **q-value** | **Shapiro-wilk test**  **for normality of residuals**  **(P value)** |
| --- | --- | --- | --- | --- | --- | --- | --- |
| 1. | Cyfra_CAT | 0.939 | 0.351 - 1.526 | 153.59 | **0.002** | **0.007** | 0.888 |
| 2. | TV+NV | 0.010 | 0.002 - 0.017 | 156.46 | **0.011** | **0.015** | 0.905 |
| 3. | NV_CAT | 0.746 | 0.136 - 1.356 | 157.40 | **0.018** | **0.022** | 0.427 |
| 4. | CRP_CAT | 0.737 | 0.125 - 1.349 | 157.04 | **0.019** | **0.028** | 0.978 |
| 5. | N | 0.427 | 0.063 - 0.791 | 157.85 | **0.023** | **0.031** | 0.530 |
| 6. | TV+NV_CAT | 0.711 | 0.098 - 1.324 | 157.96 | **0.024** | **0.035** | 0.484 |
| 7. | TV | 0.014 | 0.002 - 0.025 | 158.09 | **0.026** | **0.037** | 0.941 |
| 8. | Pain | -0.656 | -1.276 - -0.037 | 158.80 | **0.038** | **0.044** | 0.403 |
| 9. | CYFRA 21-1 | 0.059 | 0.001 - 0.117 | 159.16 | **0.047** | **0.050** | 0.782 |
| 10. | CRP | 0.038 | -0.004 - 0.080 | 159.91 | **0.072** | 0.053 | 0.587 |
| 11. | NV | 0.009 | -0.002 - 0.021 | 160.27 | **0.089** | 0.057 | 0.658 |
| 12. | Limf_CAT | -0.506 | -1.136 - 0.124 | 160.16 | 0.113 | 0.062 | 0.522 |
| 13. | 1 symptom | -0.464 | -1.098 - 0.169 | 161.11 | 0.147 | 0.068 | 0.745 |
| 14. | Sex | -0.475 | -1.186 - 0.236 | 161.48 | 0.186 | 0.077 | 0.951 |
| 15. | TtDGN_CAT | 0.411 | -0.226 - 1.047 | 161.60 | 0.201 | 0.084 | 0.852 |
| 16. | TV_CAT | 0.404 | -0.232 - 1.040 | 161.65 | 0.208 | 0.090 | 0.954 |
| 17. | TtDGN | 0.027 | -0.016 - 0.069 | 161.70 | 0.215 | 0.092 | 0.984 |
| 18. | Alb_CAT | 0.384 | -0.253 - 1.021 | 161.30 | 0.231 | 0.099 | 0.981 |
| 19. | Weigh loss | -0.471 | -1.308 - 0.366 | 162.00 | 0.263 | 0.105 | 0.978 |
| 20. | Age | 0.015 | -0.018 - 0.047 | 162.49 | 0.374 | 0.114 | 0.988 |
| 21. | WBC_CAT | -0.278 | -0.920 - 0.363 | 162.02 | 0.387 | 0.119 | 0.927 |
| 22. | > 2 symptoms | 0.393 | -0.539 - 1.326 | 162.58 | 0.401 | 0.125 | 0.949 |
| 23. | T | -0.150 | -0.519 - 0.218 | 162.62 | 0.417 | 0.130 | 0.971 |
| 24. | Neut_CAT | 0.246 | -0.396 - 0.889 | 162.70 | 0.445 | 0.136 | 0.914 |
| 25. | Neut/Lym_CAT | 0.205 | -0.439 - 0.849 | 162.38 | 0.526 | 0.141 | 0.932 |
| 26. | Neut | -0.043 | -0.224 - 0.139 | 163.09 | 0.642 | 0.147 | 0.939 |
| 27. | 2 symptoms | 0.156 | -0.567 - 0.879 | 163.12 | 0.666 | 0.152 | 0.922 |
| 28. | Limf | -0.115 | -0.651 - 0.421 | 163.12 | 0.667 | 0.156 | 0.917 |
| 29. | Plt_CAT | -0.124 | -0.769 - 0.522 | 162.65 | 0.702 | 0.161 | 0.992 |
| 30. | WBC | -0.030 | -0.188 - 0.129 | 163.17 | 0.711 | 0.163 | 0.935 |
| 31. | Neck tumor | -0.119 | -0.779 - 0.543 | 163.18 | 0.720 | 0.169 | 0.977 |
| 32. | Age_CAT | 0.087 | -0.559 - 0.733 | 163.24 | 0.787 | 0.174 | 0.990 |
| 33. | Smoking | 0.064 | -0.589 - 0.716 | 163.28 | 0.845 | 0.180 | 0.985 |
| 34. | Plt | -0.001 | -0.005 - 0.004 | 163.28 | 0.851 | 0.182 | 0.978 |
| 35. | Alb | -0.005 | -0.086 - 0.076 | 163.30 | 0.905 | 0.191 | 0.969 |
| 36. | Neut/Lym | 0.006 | -0.202 - 0.214 | 163.31 | 0.955 | 0.193 | 0.979 |
| 37. | SCC-Ag_CAT | -0.013 | -0.663 - 0.636 | 163.32 | 0.967 | 0.198 | 0.976 |
| 38. | SCC-Ag | 0.001 | -0.030 - 0.031 | 163.32 | 0.971 | 0.200 | 0.979 |

**Table S6.** Assessment of witch variables to include in multivariable regression analysis.

| **No** | **Variable** | **Significant result = 1**  **Insignificant result = 0** | | | **Score** | **Group of**  **regression analysis** |
| --- | --- | --- | --- | --- | --- | --- |
|  |  | **Correlation** | **Difference** | **Univariable regression** |  |  |
| 1. | Pain | 1 | 1 | 1 | 3 | I |
| 2. | N | 1 | 1 | 1 | 3 | I |
| 3. | NV | 1 | 1 | 0 | 2 |  |
| 4. | NV_CAT | 1 | 1 | 1 | 3 | I |
| 5. | CYFRA 21-1 | 1 | 1 | 1 | 3 |  |
| 6. | CYFRA_CAT | 1 | 1 | 1 | 3 | II |
| 7. | CRP | 0 | 1 | 0 | 1 |  |
| 8. | CRP_CAT | 1 | 1 | 1 | 3 | II |
| 9. | TV+NV | 1 | 1 | 1 | 3 |  |
| 10. | TV+NV_CAT | 1 | 1 | 1 | 3 |  |
| 11. | TV | 0 | 0 | 1 | 1 |  |
| 12. | SCC-Ag | 0 | 0 | 0 | 0 |  |
| 13. | SCC-Ag_CAT | 0 | 0 | 0 | 0 |  |
| 14. | TV_CAT | 0 | 0 | 0 | 0 |  |
| 15. | TtDGN | 0 | 0 | 0 | 0 |  |
| 16. | TtDGN_CAT | 0 | 0 | 0 | 0 |  |
| 17. | Alb | 0 | 0 | 0 | 0 |  |
| 18. | Alb_CAT | 0 | 0 | 0 | 0 |  |
| 19. | Age | 0 | 0 | 0 | 0 |  |
| 20. | Age_CAT | 0 | 0 | 0 | 0 |  |
| 21. | Smoking | 0 | 0 | 0 | 0 |  |
| 22. | Neut/Lym | 0 | 0 | 0 | 0 |  |
| 23. | Neut/Lym_CAT | 0 | 0 | 0 | 0 |  |
| 24. | Neut | 0 | 0 | 0 | 0 |  |
| 25. | Neut_CAT | 0 | 0 | 0 | 0 |  |
| 26. | Neck tumour | 0 | 0 | 0 | 0 |  |
| 27. | Limf | 0 | 0 | 0 | 0 |  |
| 28. | Limf_CAT | 0 | 0 | 0 | 0 |  |
| 29. | WBC | 0 | 0 | 0 | 0 |  |
| 30. | WBC_CAT | 0 | 0 | 0 | 0 |  |
| 31. | T | 0 | 0 | 0 | 0 |  |
| 32. | Plt | 0 | 0 | 0 | 0 |  |
| 33. | Plt_CAT | 0 | 0 | 0 | 0 |  |
| 34. | Sex | 0 | 0 | 0 | 0 |  |
| 35. | Weigh loss | 0 | 0 | 0 | 0 |  |
| 36. | 1 symptom | 0 | 0 | 0 | 0 |  |
| 37. | 2 symptoms | 0 | 0 | 0 | 0 |  |
| 38. | > 2 symptoms | 0 | 0 | 0 | 0 |  |

**Table S7.** Multivariable linear regression analysis with predictors related to tumor condition.

| **Group I: variables related to tumour condition** | | | |
| --- | --- | --- | --- |
| Full model | Summary of the model | 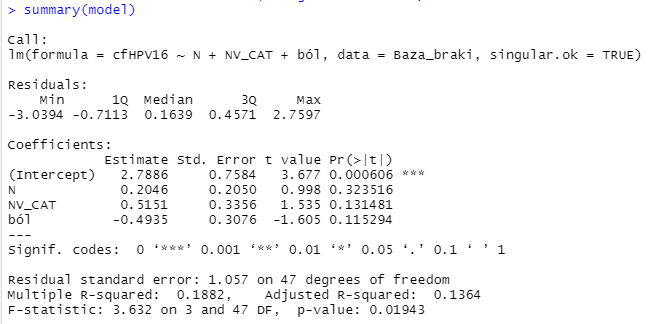 | |
|  | Plot and Test for normality of residuals | 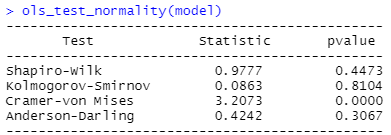 | |
|  |  | 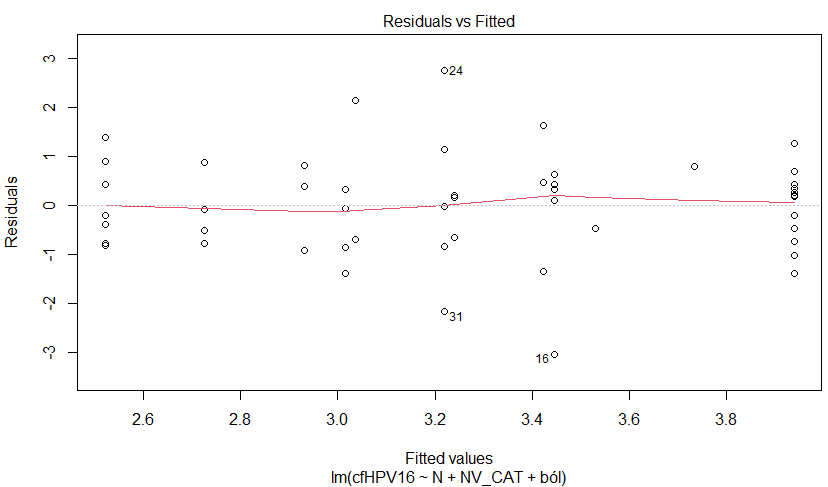 | |
| Reduced model | Summary of the model | 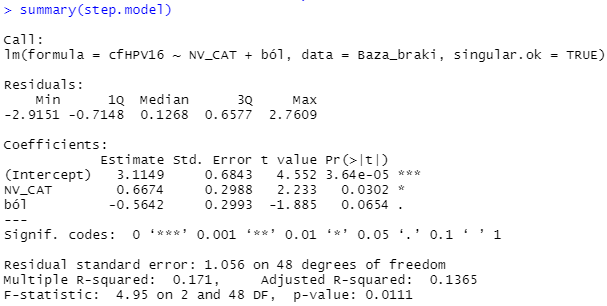 | |
|  | Plot and Test for normality of residuals | 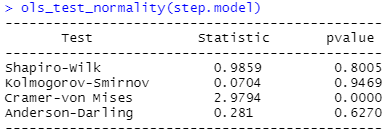 | |
|  |  | 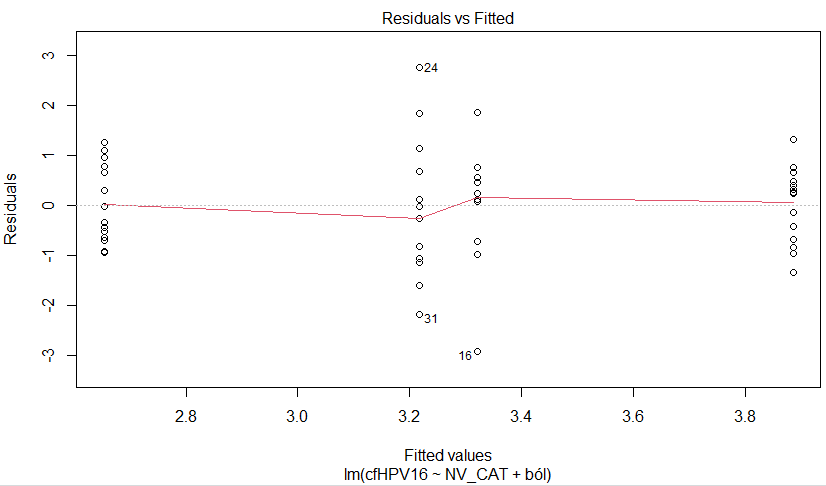 | |
| Comparison of both models on the basis of AIC | | | 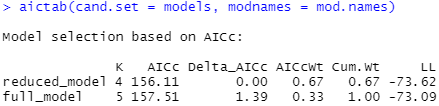 |

**Table S8.** Multivariable linear regression analysis with predictors related to biochemical diagnostics.

| **Group II: variables related to biochemical diagnostics** | | | |
| --- | --- | --- | --- |
| Full model | Summary of the model | 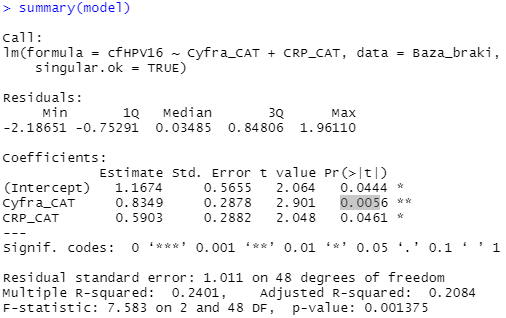 | |
|  | Plot and Test for normality of residuals | 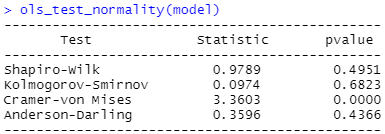 | |
|  |  | 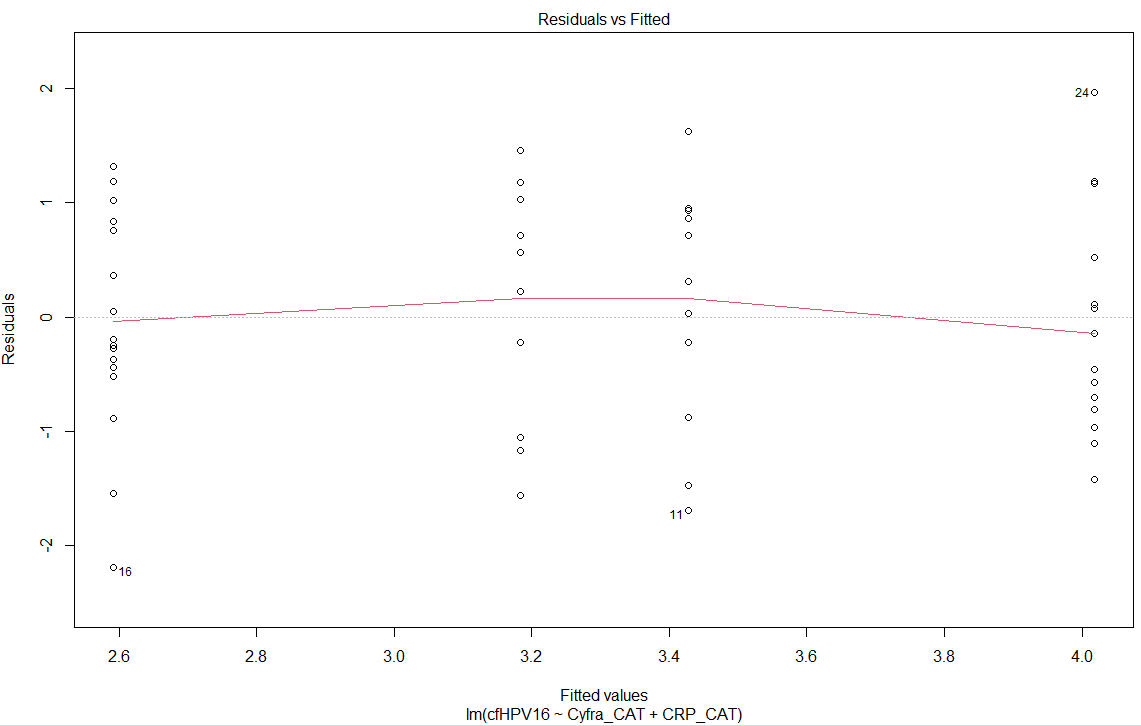 | |
| Reduced model | Summary of the model | 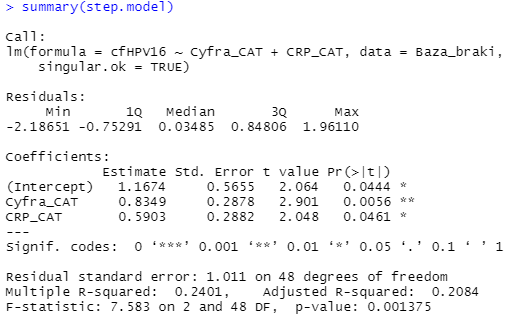 | |
|  | Plot and Test for normality of residuals | 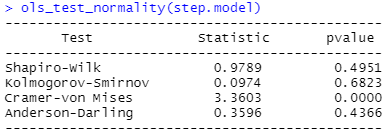 | |
|  |  | 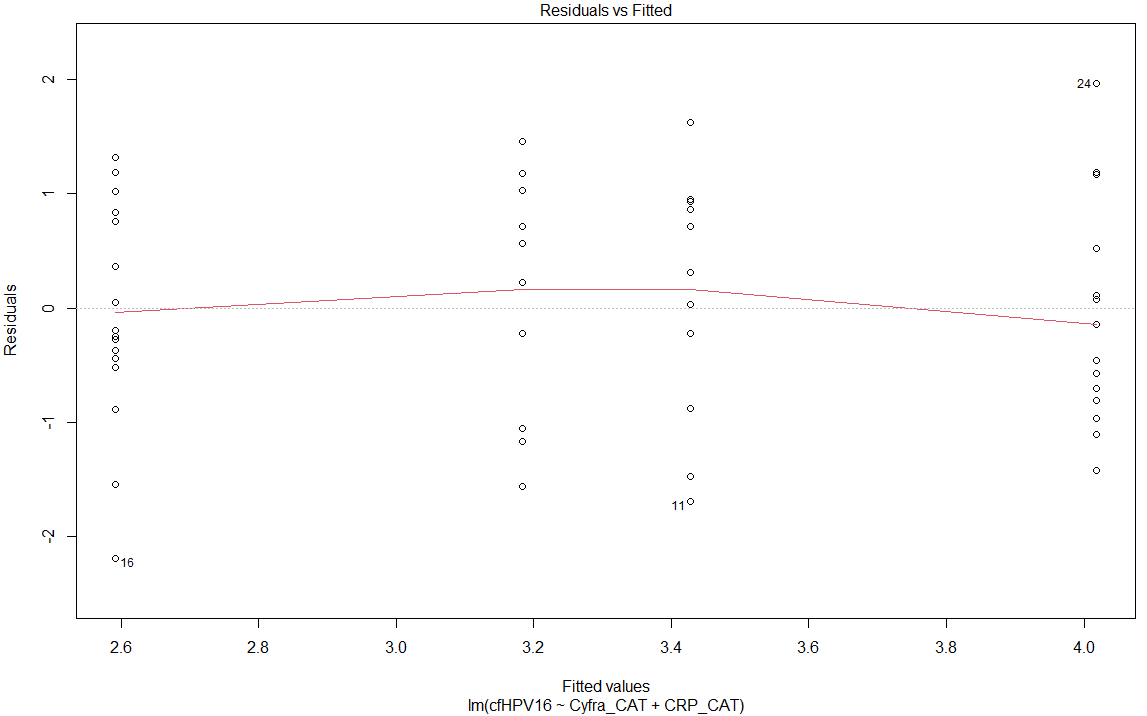 | |
| Comparison of both models on the basis of AIC | | | 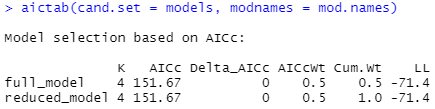 |

**Table S9.** Difference in ctHPV16 level between low and high categories of a given variable.

| **No** | **Variable** | **N** | **Mean** | **SD** | **min** | **Q1** | **Median** | **Q3** | **max** | **P value** | **q-value** | **d Cohena** |
| --- | --- | --- | --- | --- | --- | --- | --- | --- | --- | --- | --- | --- |
| 1 | TV low | 26 | 3.082 | 1.095 | 0.406 | 2.352 | 3.260 | 3.770 | 5.199 | 0.208 | 0.089 | - |
|  | TV high | 25 | 3.486 | 1.121 | 1.619 | 2.549 | 3.611 | 4.141 | 5.979 |  |  |  |
| 2 | NV low | 26 | 2.914 | 1.122 | 1.046 | 2.087 | 2.796 | 3.565 | 5.979 | **0.018** | **0.030** | **0.703** |
|  | NV high | 25 | 3.660 | 0.996 | 0.406 | 3.214 | 3.780 | 4.288 | 5.199 |  |  |  |
| 3 | TV+NV low | 26 | 2.931 | 0.988 | 1.046 | 2.087 | 3.008 | 3.574 | 5.199 | **0.024** | **0.018** | **0.665** |
|  | TV+NV high | 25 | 3.642 | 1.145 | 0.406 | 2.916 | 3.780 | 4.362 | 5.979 |  |  |  |
| 4 | CYFRA 21-1 low | 26 | 2.820 | 1.042 | 0.406 | 2.138 | 2.796 | 3.714 | 4.639 | **0.002** | **0.002** | **0.917** |
|  | CYFRA 21-1 high | 25 | 3.758 | 1.003 | 1.736 | 3.205 | 3.742 | 4.362 | 5.979 |  |  |  |

**Table S10.** The difference in the mean value of initial ctHPV16 VL for various clinical factors. Results of univariable analysis.

| **Variable** | **Category** | **Total number**  **of patients (N = 51)** | **ctHPV16**  **Mean (SD)** | **P value** | | **q-value** | | **Cohen's**  **d** |
| --- | --- | --- | --- | --- | --- | --- | --- | --- |
| sex | Males  Females | 37  14 | 3.410 (0.982)  2.935 (1.380) | 0.186 | | 0.073 | | - |
| Age | <= 57  > 57 | 26  25 | 3.237 (0.406)  3.324 (1.046) | 0.787 | | 0.171 | | - |
| Smokers | Yes  No | 22  29 | 3.316 (1.096)  3.252 (1.147) | 0.845 | | 0.176 | | - |
| Symptoms | Pain Yes  Pain No | 24  27 | 2.932 (1.012)  3.589 (1.132) | **0.038** | | **0.040** | | **0.613** |
|  | Neck tumor Yes  Neck tumor No | 31  20 | 3.233 (1.075)  3.352 (1.197) | 0.720 | | 0.165 | | - |
|  | Weight loss Yes  Weight loss No | 9  42 | 2.892 (0.827)  3.363 (1.163) | 0.263 | | 0.101 | | - |
|  | 1 symptom  > 1 symptom | 27  24 | 3.498 (1.056)  3.034 (1.151) | 0.147 | | 0.064 | | - |
|  | 2 symptoms  > 2 symptoms | 14  37 | 3.166 (1.265)  3.323 (1.065) | 0.666 | | 0.149 | | - |
|  | > 2 symptoms  <= 2 symptoms | 7  44 | 2.940 (0.790)  3.334 (1.161) | 0.401 | | 0.121 | | - |
| Symptoms duration  (months) | <= 5  > 5 | 27  24 | 3.086 (1.073)  3.497 (1.144) | 0.201 | | 0.081 | | - |
| SCC  (median) | <= 1.9  > 1.9 | 29  22 | 3.286 (1.117)  3.272 (1.136) | 0.967 | | 0.194 | | - |
| CYFRA 21-1  (median) | CYFRA 21-1 low <= 3.75  CYFRA 21-1 high > 3.75 | 26  25 | 2.820 (1.042)  3.758 (1.003) | **0.002** | | **0.002** | | **0.917** |
| T | 1  2  3  4 | 5  22  16  8 | 3.482 (1.053)  3.364 (0.948)  3.221 (1.158)  3.039 (1.457) | 0.884 | | 0.189 | | - |
| N | 1  2  3 | 14  13  24 | 2.774 (0.954)  3.184 (1.229)  3.626 (1.035) | 1 vs. 2: 0.301 | **0.076** | 0.108 | 0.055 | - |
|  |  |  |  | 1 vs. 3: **0.044** |  | **0.046** |  | **0.857** |
|  |  |  |  | 2 vs. 3: 0.264 |  | 0.106 |  | - |
| TV  (cm^3^) | TV low <= 32.457  TV high > 32.457 | 26  25 | 3.082 (1.095)  3.486 (1.121) | 0.208 | | 0.086 | | - |
| NV  (cm^3^) | NV low <= 26.92  NV high > 26.92 | 26  25 | 2.914 (1.122)  3.660 (0.996) | **0.023** | | **0.029** | | **0.703** |
| TV+NV  (cm^3^) | TV+NV low <= 60.54  TV+NV high > 60.54 | 26  25 | 2.931 (0.988)  3.642 (1.145) | **0.018** | | **0.018** | | **0.827** |
| CRP  (median) | CRP low <= 2.03  CRP high > 2.03 | 27  24 | 2.933 (1.096)  3.670 (1.027) | **0.019** | | **0.024** | | **0.694** |
| Alb  (median) | Alb low <= 43  Alb high > 43 | 26  25 | 3.091 (1.293)  3.476 (0.878) | 0.231 | | 0.095 | |  |
| Neut/Lym  (median) | Neut/Lym low <= 2.252  Neut/Lym high > 2.252 | 26  25 | 3.179 (1.064)  3.384 (1.178) | 0.526 | | 0.138 | |  |
| Neut  (median) | Neut low <= 4.3  Neut high > 4.3 | 26  25 | 3.159 (1.149)  3.405 (1.087) | 0.445 | | 0.132 | |  |
| Limf  (median) | Limf low <= 1.89  Limf high > 1.89 | 26  25 | 3.528 (1.221)  3.022 (0.951) | 0.113 | | 0.059 | |  |
| WBC  (median) | WBC low <= 6.99  WBC high > 6.99 | 26  25 | 3.416 (1.260)  3.138 (0.945) | 0.387 | | 0.116 | |  |
| Plt  (median) | Plt low <= 259.000  Plt high > 259.000 | 26  25 | 3.340 (1.158)  3.217 (1.088) | 0.702 | | 0.158 | |  |

**Table S11.** Multivariable linear regression analysis with predictors related to tumor condition.

| **Full model** | | | | **Reduced model** | | | | |
| --- | --- | --- | --- | --- | --- | --- | --- | --- |
| **Variable** | **Estimate** | **95% CI** | **P value** | **Variable** | **Estimate** | **95% CI** | **P value** | **q-value** |
| N | 0.205 | -0.208 - 0.617 | 0.324 |  |  |  |  |  |
| NV_CAT | 0.515 | -0.160 - 1.190 | 0.132 | NV_CAT | 0.667 | 0.067 - 1.268 | **0.030** | **0.039** |
| Pain | -0.494 | -1.112 - 0.125 | 0.115 | Pain | -0.564 | -1.166 - 0.038 | 0.065 | 0.051 |

*NV_CAT - nodal volume categorised below and over median value, N - N-stage acc to AJCC 8^th^.

**Table S12.** Multivariable linear regression analysis with predictors related to biochemical factors.

| **Full model** | | | | **Reduced model **** | | | | |
| --- | --- | --- | --- | --- | --- | --- | --- | --- |
| **Variable** | **Estimate** | **95% CI** | **P value** | **Variable** | **Estimate** | **95% CI** | **P value** | **q-value** |
| CYFRA_CAT | 0.835 | 0.256 - 1.414 | **0.006** | CYFRA_CAT | 0.835 | 0.256 - 1.414 | **0.006** | **0.011** |
| CRP_CAT | 0.590 | 0.0108 - 1.170 | **0.046** | CRP_CAT | 0.590 | 0.0108 - 1.170 | **0.046** | **0.048** |

*CYFRA_CAT - level of CYFRA 21-1 categorised below and over median value, CRP_CAT - C-Reactive Protein concentration categorised below and over median value. ** full model could not be reduced further to get a better model.
